# Association between triglyceride glucose–body mass index and clinical outcomes after septal myectomy

**DOI:** 10.3389/fendo.2026.1855510

**Published:** 2026-07-30

**Authors:** Changpeng Song, Xinxin Zheng, Jingang Cui, Xiaohong Huang, Qiulan Yang, Shuiyun Wang

**Affiliations:** 1 Department of Special Medical Treatment Center, State Key Laboratory of Cardiovascular Disease, Fuwai Hospital, National Center for Cardiovascular Diseases, Chinese Academy of Medical Sciences and Peking Union Medical College, Beijing, China; 2 Department of Cardiovascular Surgery, State Key Laboratory of Cardiovascular Disease, Fuwai Hospital, National Center for Cardiovascular Diseases, Chinese Academy of Medical Sciences and Peking Union Medical College, Beijing, China; 3 Department of Cardiovascular Surgery, Fuwai Shenzhen Hospital, Chinese Academy of Medical Sciences, Shenzhen, China

**Keywords:** hypertrophic obstructive cardiomyopathy, insulin resistance, outcomes, septal myectomy, TyG-BMI

## Abstract

**Aims:**

This study examined the association between triglyceride glucose-body mass index (TyG–BMI) and clinical outcomes in patients with hypertrophic obstructive cardiomyopathy (HOCM) undergoing septal myectomy.

**Methods:**

This retrospective cohort study was conducted at Fuwai Hospital, including 1,302 patients with HOCM who underwent septal myectomy. According to the tertile distribution of TyG–BMI, the participants were classified into three groups (T1–T3). The primary endpoint consisted of heart failure hospitalization or all-cause death.

**Results:**

The mean age of the participants was 49.2 ± 11.8 years, of whom 61% (791) were men. During a median follow-up of 4.3 (3.1–6.2) years, the cumulative incidences of primary endpoint and all-cause death differed significantly across the three groups on the Kaplan–Meier analysis (log-rank *P* < 0.05). Cox analysis showed that patients in group T3 exhibited the greatest incidence rates of primary endpoint (adjusted hazard ratio (HR) = 2.00, 95% confidence interval (CI) 1.16–3.44, *P* = 0.012) as well as the highest risk of all-cause death (adjusted HR = 3.39, 95% CI 1.34–8.58, *P* = 0.010) compared to those in group T1. When treated as a continuous variable, TyG–BMI remained significantly linked to an elevated risk of primary endpoint (adjusted HR = 1.007, 95% CI 1.002–1.012, *P* = 0.009) and increased all-cause death (adjusted HR = 1.013, 95% CI 1.006–1.021, *P* = 0.001).

**Conclusions:**

Higher TyG–BMI was associated with an increased risk of adverse long-term outcomes after septal myectomy in patients with HOCM.

## Introduction

Hypertrophic cardiomyopathy (HCM) can lead to myocardial ischemia, sudden cardiac death, and heart failure (1, 2). For patients with symptoms refractory to medical management and a left ventricular outflow tract (LVOT) gradient of 50 mmHg or higher, surgical myectomy remains the gold standard therapeutic intervention to relieve obstruction and improve long-term survival (1–3). Although surgical techniques have continued to improve and pharmacologic therapy has advanced (4), long-term postoperative prognosis remains a clinical concern. Identifying potential risk factors for adverse outcomes and implementing early intervention are of great importance.

Metabolic dysfunction is an important factor influencing myocardial structure and function. Insulin resistance (IR) refers to diminished tissue sensitivity to normal insulin concentrations, which is linked to higher incidence of type 2 diabetes mellitus (DM) and cardiovascular disorders (5). There are multiple methods to assess IR (6). The triglyceride glucose index (TyG) has been shown to be a simple surrogate marker of IR (7). Several TyG-derived indices have recently been reported (7). Triglyceride glucose–body mass index (TyG–BMI) has been proposed as a practical surrogate marker that combines lipid–glucose metabolism and adiposity (8). Furthermore, elevated TyG–BMI has been identified as a risk factor for adverse outcomes in many cardiovascular disorders (7, 9, 10).

However, evidence regarding the relationship between TyG–BMI and clinical outcomes after septal myectomy in hypertrophic obstructive cardiomyopathy (HOCM) is scarce. Therefore, the present study aimed to assess the relationship between TyG–BMI and postoperative adverse outcomes in patients undergoing septal myectomy.

## Materials and methods

### Study design and participants

This retrospective cohort study was reported in accordance with the STROBE recommendations for observational studies. A STROBE-style flow diagram was used to describe patient screening, exclusion criteria, the final analytic cohort, and the distribution of patients across TyG–BMI tertiles.

**Figure 1:**
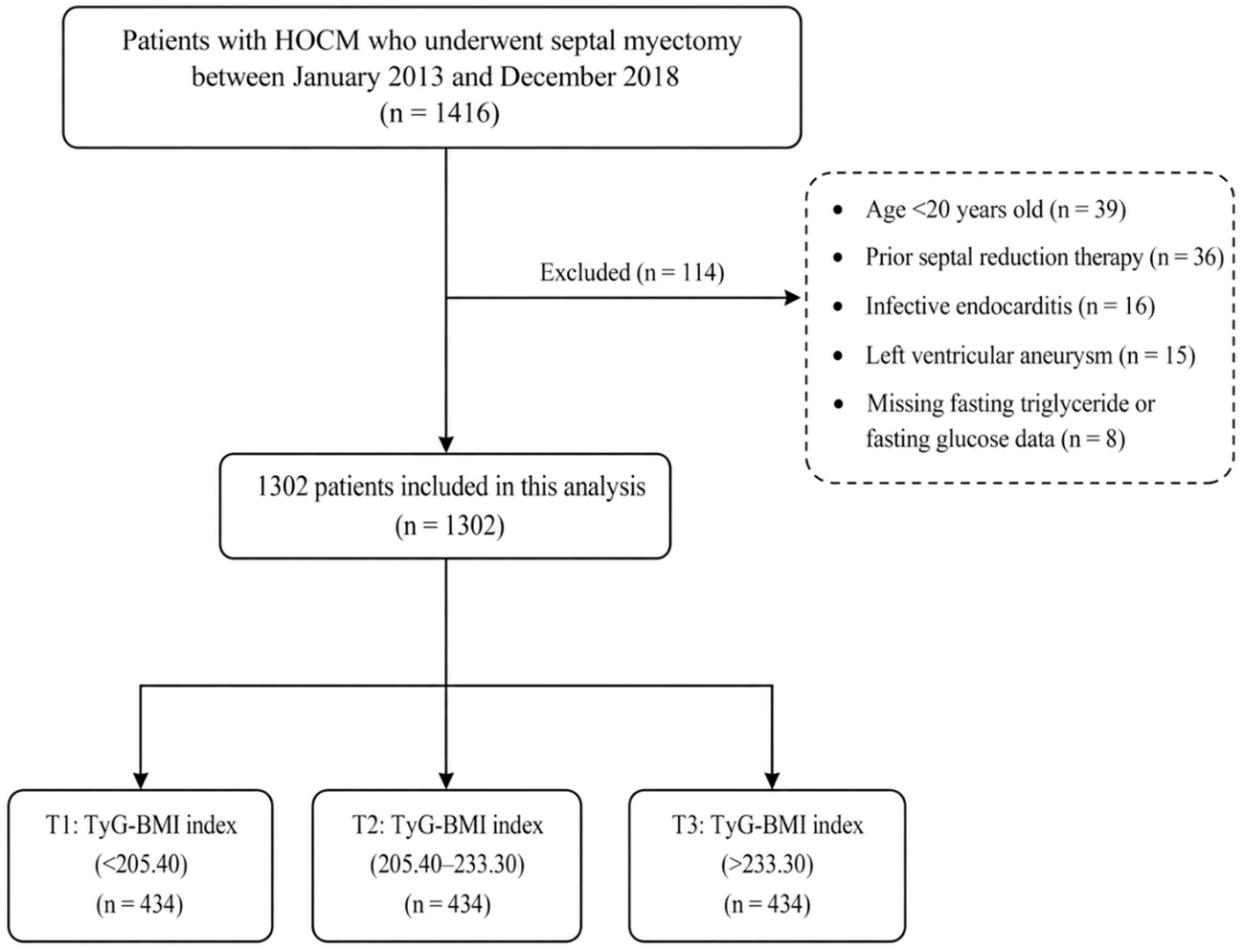
Flowchart of patient selection. The flowchart shows the selection process of patients included in the final analysis.

We screened 1,416 HOCM patients with available follow-up information who underwent septal myectomy at Fuwai Hospital between January 2013 and December 2018. The diagnosis of HOCM and the selection of candidates for septal myectomy were based on established guideline criteria. The exclusion criteria included age younger than 20 years, prior septal reduction therapy, infective endocarditis, and left ventricular aneurysm. Patients with missing fasting triglyceride or fasting glucose data were also excluded because these variables were required for the calculation of TyG–BMI. The final analysis was therefore performed in patients with complete data for TyG–BMI calculation. Baseline demographics and clinical characteristics were extracted from the medical records. Ultimately, 1,302 patients were included in the final analysis (**Figure 1**).

### TyG–BMI calculation

TyG–BMI was calculated as TyG multiplied by BMI. BMI was calculated as weight divided by height squared (kg/m^2^): TyG = ln [fasting triglycerides (mg/dl) × fasting glucose (mg/dl)/2]. On the basis of TyG–BMI tertiles, the cohort was stratified into three groups: group T1 (*n* = 434, TyG–BMI < 205.40), group T2 [*n* = 434, TyG–BMI (205.40–233.30)], and group T3 (*n* = 434, TyG–BMI > 233.30).

### Definition of adverse outcomes

The primary endpoint was analyzed as time to first event of the composite outcome, defined as heart failure hospitalization or all-cause death, while all-cause death was analyzed separately as the secondary endpoint. Heart failure hospitalization was defined as an unplanned hospitalization primarily due to worsening heart failure, supported by clinical symptoms and/or signs of heart failure and requiring heart-failure-specific treatment during hospitalization. Patients who did not experience either event were censored at the date of last available follow-up. Follow-up data were obtained via outpatient clinic evaluation or telephone interviews.

### Statistical analysis

Continuous variables were presented as mean ± SD or median (interquartile), as appropriate, according to their distribution. ANOVA test and Mann–Whitney *U*-test were conducted to test differences across groups accordingly. Categorical variables were expressed as number (percentage). Chi-square test or Fisher’s exact test was performed to test the differences across groups appropriately. We performed a comparison of cumulative incidence of adverse outcomes across the three groups using Kaplan–Meier curve analysis. The proportional hazards assumption was assessed using Schoenfeld residuals with the cox.zph function in the survival package in R (**Supplementary Table 1**). Cox proportional hazards models were constructed to evaluate the prognostic value of TyG–BMI on clinical outcomes. Model 2 adjusted for sex and age, whereas model 3 adjusted for sex, age, and variables with *P <*0.05 in univariable Cox regression analysis. In both adjusted models, group T1 was used as the reference. Additionally, stratified Cox hazards models were performed for subgroup assessment. A two-sided *P*-value <0.05 was considered statistically significant. GraphPad Prism 9.0 (GraphPad), SPSS 25.0 (IBM Corp), and R software (version 4.4.3) were used in this study.

## Results

### Baseline data of study population

A total of 1,302 patients were included in the final analytic cohort and classified into three equal groups according to TyG–BMI tertiles. The average age was 49.2 ± 11.8 years. Of the participants, 791 (61%) were men. The mean BMI was 25.6 ± 3.5 kg/m^2^. Several parameters differed significantly across tertiles, including age, sex, BMI, triglycerides, fasting blood glucose, hypertension, diabetes mellitus, systolic and diastolic blood pressure, LAD, LVEDD, LVEF, posterior wall thickness, right ventricular dimension, and moderate to severe mitral regurgitation (details are shown in **Table 1**).

**Table 1:** Demographic and clinical characteristics of the study participants.

Characteristics	T1 (<205.40)	T2 (205.40–233.30)	T3 (>233.30)	*P*-value
*N*	434	434	434	
Age (years)	46.9 ± 13.1	50.7 ± 11.5	50.1 ± 10.4	<0.001
Sex (*n* male/*n* female)	221/213	281/153	289/145	<0.001
Heart rate (bpm)	72.4 ± 10.0	72.1 ± 9.8	72.3 ± 9.4	0.898
Systolic blood pressure (mmHg)	117.7 ± 14.4	123.0 ± 15.6	124.9 ± 15.8	<0.001
Diastolic blood pressure (mmHg)	69.9 ± 9.6	73.4 ± 9.8	74.5 ± 9.7	<0.001
BMI (kg/m^2^)	22.1 ± 2.1	25.7 ± 1.4	29.1 ± 2.5	<0.001
Fasting glucose (mg/dL)	83.2 ± 12.5	88.3 ± 14.2	97.6 ± 58.0	<0.001
Triglyceride (mg/dL)	97.1 ± 40.0	127.4 ± 52.1	182.4 ± 122.3	<0.001
TyG index	8.2 ± 0.4	8.5 ± 0.4	8.9 ± 0.5	<0.001
Hypertension, *n* (%)	86 (20)	136 (31)	191 (44)	<0.001
Atrial fibrillation, *n* (%)	66 (15)	58 (13)	69 (16)	0.572
Diabetes mellitus, *n* (%)	10 (2)	20 (5)	47 (11)	<0.001
NYHA III/IV, *n* (%)	312 (72)	314 (72)	317 (73)	0.936
Echocardiographic data				
LAD (mm)	43.2 ± 7.3	45.1 ± 6.9	45.6 ± 6.9	<0.001
LVEDD (mm)	40.7 ± 5.3	43.3 ± 5.2	44.0 ± 5.0	<0.001
LVEF (%)	71.1 ± 6.2	69.9 ± 6.0	69.6 ± 5.8	<0.001
MWT (mm)	22.7 ± 4.9	22.5 ± 5.0	22.5 ± 4.8	0.755
PWT (mm)	11.6 ± 2.8	11.8 ± 2.4	12.2 ± 2.5	0.005
Maximum LVOTG (mmHg)	85.8 ± 30.2	85.9 ± 29.5	81.8 ± 27.8	0.056
RVD (mm)	20.2 ± 3.2	21.2 ± 3.2	21.6 ± 2.9	<0.001
Moderate to severe MR, n (%)	258 (59)	235 (54)	214 (49)	0.011

BMI, body mass index; MWT, maximum wall thickness; LAD, left atrial dimension; LVEDD, left ventricular end-diastolic dimension; LVEF, left ventricular ejection fraction; LVOTG, left ventricular outflow tract gradient; MR, mitral regurgitation; NYHA, New York Heart Association; PWT, posterior wall thickness; RVD, right ventricular dimension.

### Intraoperative and postoperative outcomes

Concomitant procedures were common in patients who underwent septal myectomy, including coronary artery bypass grafting (CABG), valvular procedures, myocardial unroofing, and surgical ablation. The proportions of CABG and tricuspid valve procedures differed significantly across the three groups. A higher TyG–BMI was associated with shorter ventilation time, intensive care unit stays, and postoperative hospital stays (all *P*-values <0.05) (**Table 2**).

**Table 2:** Intraoperative and postoperative outcomes.

Characteristics	T1 (*n* = 434)	T2 (*n* = 434)	T3 (*n* = 434)	*P*-value
Aortic valve procedure	8 (2)	12 (3)	9 (2)	0.632
Mitral valve procedure	62 (14)	53 (12)	48 (11)	0.347
Tricuspid valve procedure	4 (1)	23 (5)	10 (2)	<0.001
Myocardial unroofing	20 (5)	22 (5)	16 (4)	0.603
CABG	48 (11)	67 (15)	86 (20)	0.002
Maze ablation	25 (6)	27 (6)	26 (6)	0.960
CPB time, min	90 (75–115)	94 (76–120)	94 (78–122)	0.429
Aortic clamp time, min	60 (49–81)	62 (50–81)	62 (50–81)	0.406
Ventilation, h	18 (14–20)	17 (14–20)	16 (13–19)	<0.001
ICU stay, h	48 (24–72)	46 (24–72)	30 (24–60)	0.008
Postoperative length of stay, days	7 (6–9)	7 (7–9)	7 (6–8)	0.015

CABG, coronary artery bypass grafting; CPB, cardiopulmonary bypass; ICU, intensive care unit.

### Clinical outcomes during follow-up

**Figure 2:**
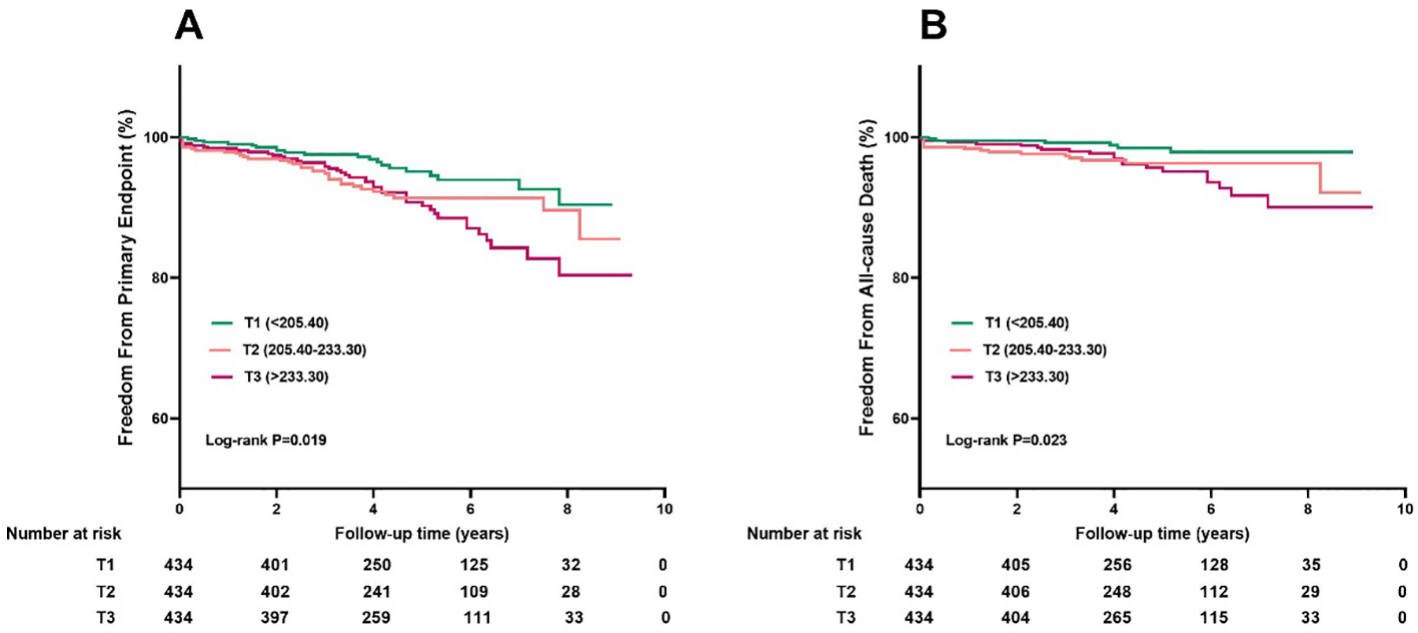
Kaplan–Meier curves for outcomes after septal myectomy. **(A)** Primary endpoint, defined as all-cause death or heart failure hospitalization. **(B)** All-cause death.

Across a median follow-up duration of 4.3 (3.1–6.2) years, 95 patients experienced the primary endpoint, including 41 all-cause deaths and 54 heart failure hospitalizations. The numbers of primary endpoint events across the low, middle, and high TyG–BMI tertiles were 20, 33, and 42, respectively. The corresponding numbers of all-cause deaths were six, 15, and 20, and the numbers of heart failure hospitalizations were 14, 18, and 22, respectively. The Kaplan–Meier analysis showed a distinct incidence of primary endpoint and all-cause death across the three groups (**Figure 2**). In comparison with those in group T1, patients in group T3 with higher TyG–BMI had the highest rates of primary endpoint and all-cause death (log-rank *P* = 0.019 for primary endpoint and *P* = 0.023 for all-cause death).

### Association of TyG–BMI with adverse outcomes

We performed Cox regression analyses to investigate the influence of TyG–BMI on adverse outcomes. In model 3, the primary endpoint model was adjusted for age, sex, and variables with *P <*0.05 in univariable Cox regression analysis (**Supplementary Table 2**). Patients in group T3 had the highest risk of primary endpoint [hazard ratio (HR) = 2.00, 95% confidence interval (CI) 1.16–3.44, *P* = 0.012] as well as all-cause death (HR = 3.39, 95% CI 1.34–8.58, *P* = 0.010) compared to those in group T1. When TyG–BMI was entered into the model as a continuous variable, elevated TyG–BMI remained significantly correlated with increased risk of primary endpoint (adjusted HR = 1.007, 95% CI 1.002–1.012, *P* = 0.009) and all-cause death (adjusted HR = 1.013, 95% CI 1.006–1.021, *P* = 0.001) even after adjustment for the selected potential confounders (**Table 3**).

**Table 3:** Univariable and adjusted Cox regression analysis of the relation between TyG–BMI and clinical outcomes.

	Model 1		Model 2^a^		Model 3^b^	
	HR (95% CI)	*P*-value	HR (95% CI)	P-value	HR (95% CI)	P-value
Composite endpoint						
TyG–BMI	1.007 (1.002–1.013)	0.004	1.007 (1.002–1.012)	0.011	1.007 (1.002–1.012)	0.009
T1	Reference		Reference		Reference	
T2	1.70 (0.98–2.97)	0.060	1.54 (0.88–2.71)	0.133	1.60 (0.91–2.82)	0.105
T3	2.12 (1.24–3.61)	0.006	1.97 (1.15–3.38)	0.014	2.00 (1.16–3.44)	0.012
All-cause death						
TyG–BMI	1.012 (1.005–1.019)	<0.001	1.012 (1.005–1.021)	0.001	1.013 (1.006–1.021)	0.001
T1	Reference		Reference		Reference	
T2	2.59 (1.01–6.68)	0.049	2.31 (0.89–6.02)	0.086	2.52 (0.96–6.59)	0.059
T3	3.37 (1.35–8.38)	0.009	3.06 (1.22–7.71)	0.017	3.39 (1.34–8.58)	0.010

^a^Model 2 was adjusted for age and sex.

^b^Model 3 was adjusted for age, sex, left atrial dimension, and atrial fibrillation for the composite endpoint and for age, sex, atrial fibrillation, and left ventricular outflow tract gradient for all-cause mortality.

### Subgroup analyses

Exploratory subgroup analyses were performed to evaluate whether the association between TyG–BMI and long-term outcomes was generally consistent across clinically relevant strata, including age, sex, BMI, DM, hypertension, LAD, and maximum wall thickness. Overall, higher TyG–BMI showed a generally consistent association with the composite endpoint in most subgroups. A statistically significant interaction was observed for hypertension status. Similarly, higher TyG–BMI was associated with all-cause mortality in most subgroups. Significant interaction terms were observed for age, BMI, hypertension, and maximum wall thickness. However, this finding should be interpreted cautiously because the number of events in some strata was limited (details are shown in **Table 4**).

**Table 4:** Subgroup analyses of the association between TyG–BMI and clinical outcomes in patients undergoing septal myectomy.

Characteristics	Number of participants	Composite endpoint			All-cause death		
		HR (95% CI)	*P*-value	*P* for interaction	HR (95% CI)	*P*-value	*P* for interaction
Age (years)				0.329			0.047
<60	1,016	1.009 (1.003–1.015)	0.003		1.016 (1.009–1.023)	<0.001	
≥60	286	1.003 (0.993–1.013)	0.561		0.998 (0.982–1.015)	0.830	
Sex				0.068			0.054
Male	791	1.003 (0.996–1.010)	0.358		1.012 (1.003–1.020)	0.010	
Female	511	1.013 (1.005–1.020)	0.001		1.013 (1.001–1.025)	0.028	
BMI (kg/m^2^)				0.762			0.030
<30	1,170	1.011 (1.003–1.018)	0.005		1.019 (1.007–1.031)	0.002	
≥30	132	1.006 (0.99–1.023)	0.452		1.021 (1.003–1.039)	0.024	
Diabetes mellitus				0.457			0.123
No	1,225	1.009 (1.003–1.014)	0.002		1.016 (1.008–1.024)	<0.001	
Yes	77	1.002 (0.983–1.021)	0.833		0.993 (0.965–1.021)	0.623	
Hypertension				0.047			0.026
No	889	1.011 (1.005–1.017)	<0.001		1.017 (1.010–1.024)	<0.001	
Yes	413	0.999 (0.990–1.009)	0.892		0.998 (0.982–1.013)	0.770	
Left atrial dimension (mm)				0.378			0.770
<45	669	1.009 (1.003–1.016)	0.006		1.013 (1.004–1.023)	0.007	
≥45	633	1.005 (0.997–1.012)	0.227		1.011 (1.000–1.021)	0.041	
Maximum wall thickness (mm)				0.109			0.006
<30	1,186	1.005 (1.000–1.011)	0.063		1.006 (0.998–1.015)	0.159	
≥30	116	1.015 (1.005–1.024)	0.002		1.026 (1.014–1.039)	<0.001	

HR, hazard ratio; 95% CI, 95% confidence interval.

## Discussion

In this retrospective cohort study, the association between TyG–BMI and adverse prognostic events was evaluated in patients with HOCM who underwent septal myectomy. The results showed that higher TyG–BMI was significantly associated with higher risk of primary endpoint and all-cause death.

IR is a hallmark of metabolic dysfunction and is associated with the progression and prognosis of cardiovascular disease (9–11). Previous data showed that IR is independently associated with elevated rates of mortality and hospitalization in patients with heart failure (10). Metabolic disturbance may contribute to this process, as it can result in myocardial energy metabolism impairment, endothelial dysfunction, and increased inflammation. These long-standing pathophysiological changes subsequently cause myocardial injury and cardiac remodeling (12, 13). Considering that HCM is characterized by marked myocardial hypertrophy, the impact of IR on the promotion of myocardial hypertrophy and fibrosis could be more striking. Moreover, subjects with both HF and HCM and comorbid IR are more likely to suffer arrhythmias, SCD, and heart failure progression (14, 15).

Several indices are available to evaluate insulin resistance. The TyG index is widely regarded as a robust marker reflecting IR, which is highly correlated to HOMA-IR. The TyG–BMI incorporates the effects of both TyG and BMI, which can provide a more comprehensive assessment of metabolic status (6, 7). This combined index has shown better predictive value for cardiovascular diseases than TyG alone. While there is limited research directly linking TyG–BMI to clinical features and prognosis among patients with HCM, existing studies on TyG index and BMI provide valuable insights. A previous study showed that TyG index was associated with greater LVEDD in HCM (16). In this cohort, TyG–BMI was also positively associated with LVEDD. Additionally, left atrial dimension, posterior wall thickness, LVEF, and right ventricular dimension were also significantly different across among the three groups. Considering that obesity is associated with IR (17) and a significant factor influencing cardiac structure and function in HCM (18), the discrepancy between TyG index and TyG–BMI regarding echocardiographic characteristics might be partially attributed to the additional effect of BMI. Therefore, TyG–BMI, by accounting for both metabolic and adiposity factors, may provide additional information about metabolic and adiposity-related status in HCM patients.

In terms of prognosis, a prior study reported an inverse association between TyG and clinical outcomes in individuals with HOCM treated with septal reduction therapy (19). However, our investigation observed that patients with higher TyG–BMI values experienced worse long-term outcomes. Notably, the previous study excluded the patients with DM (19), whereas patients with DM were included in the present analysis. Several large-scale studies have reported that DM exerts a significant influence on cardiac remodeling and long-term survival in HCM (20, 21). In addition, the treatment strategies and endpoints differed between studies. The prior study (19) included both invasive and non-invasive treatment groups and used cardiogenic death as the primary endpoint, whereas our study focused on patients undergoing surgical septal myectomy and used a composite endpoint of all-cause death or heart failure hospitalization. Furthermore, as noted previously, the TyG–BMI incorporates BMI-related metabolic effects. Obesity is well established as a major risk factor in various cardiovascular diseases including HCM (18, 22). These differences could partially explain the discrepant findings. The subgroup analyses suggested significant interactions between TyG–BMI and hypertension, age, and wall thickness. These findings indicate that the association between TyG–BMI and adverse outcomes may vary across clinically relevant subgroups. The relative contribution of TyG–BMI to mortality risk is attenuated in older patients possibly because of competing risks, accumulated comorbidities, frailty, and other age-related non-metabolic factors. In addition, TyG–BMI lost statistical significance for the composite endpoint among patients with hypertension. Hypertension itself is a major risk factor for cardiovascular disease. In patients with HOCM, pressure overload, dynamic LVOT obstruction, and microvascular dysfunction can interact with systemic vascular tone to aggravate myocardial hypertrophy and diastolic dysfunction. Antihypertensive therapy may modify vascular tone, myocardial wall stress, and metabolic status; therefore, heterogeneity in hypertension severity and antihypertensive treatment could attenuate or mask the observed association between TyG–BMI and clinical outcomes in hypertensive patients. The exact mechanisms underlying these observations are not yet fully understood, but several potential pathways may contribute. The TyG–BMI incorporates both metabolic and adiposity factors, providing a more in-depth evaluation of IR, and may reflect a higher-risk metabolic phenotype in HCM. Additionally, obesity contributes to IR and increases cardiovascular risk. TyG–BMI incorporates obesity-related information into the assessment of insulin resistance. This combination may increase the predictive accuracy for worse outcomes in HCM patients, as obesity may contribute to the progression of HCM. In addition, IR and obesity have been linked to inflammation and altered metabolism, which are also associated with myocardial fibrosis and hypertrophy (23, 24).

Our study highlighted the potential relevance of TyG–BMI for preoperative risk assessment and postoperative metabolic management in HOCM. The elevation in the TyG–BMI corresponded to worse clinical outcomes. Given its significant relationship with clinical outcomes, TyG–BMI may serve as an accessible adjunctive marker of metabolic risk, but its clinical utility requires external validation. Furthermore, our findings highlighted the potential benefit of long-term management of IR and metabolic dysfunction. This finding is consistent with another study that reported that metabolic alterations can influence cardiac function in HCM (25).

### Study limitations

Several limitations should be noted. First, as a retrospective observational study, this analysis was subject to inherent selection bias and residual confounding. Although adjusted for many variables, we believe that there are several unmeasured factors, such as genetic background, lifestyle factors, medication use, and postoperative management, that may have influenced the observed associations. Second, baseline medication data were not systematically obtained in the retrospective surgical database. Lipid-lowering drugs, glucose-lowering agents, and antihypertensive medications may influence triglycerides, fasting glucose, body weight, blood pressure, vascular tone, and cardiac remodeling. The absence of these medication variables may have introduced medication-related confounding. Third, only preoperative baseline TyG–BMI values were available. Septal myectomy, postoperative recovery, changes in symptoms and exercise capacity, lifestyle modification, and subsequent medical treatment may alter metabolic status during follow-up. Because longitudinal TyG–BMI measurements were not available, we could not evaluate whether dynamic metabolic trajectories after surgery were associated with cardiac remodeling or late clinical outcomes. Fourth, waist circumference, waist-to-hip ratio, and waist-to-height ratio were not systematically collected in the present cohort. Therefore, we were unable to assess whether central-adiposity-based indices, such as TyG-WC or TyG-WHR, would provide additional prognostic information beyond TyG–BMI. Fifth, because this study included only patients undergoing septal myectomy, the findings may not be generalizable to all patients with HOCM.

## Conclusion

Higher TyG–BMI was associated with adverse long-term outcomes after septal myectomy in patients with HOCM. Its incremental prognostic value and clinical utility require validation in prospective external cohorts.

## Data Availability

Data are available from the corresponding author upon reasonable request. Requests to access the datasets should be directed to Changpeng Song (SongChp-FW@outlook.com).
