# Supplementary material for: Association between triglyceride glucose–body mass index and clinical outcomes after septal myectomy
Source: Front Endocrinol (Lausanne). 2026 Jul 30;17:1855510. doi: 10.3389/fendo.2026.1855510 (PMC13467838; doi:10.3389/fendo.2026.1855510)
Supplement: Supplementary file 1 [file Table1.docx]

**Table S1. Proportional hazards assumption tests for the initial and sex-stratified Cox models**

| **Outcome** | **Cox model** | **Variable** | **χ²** | **df** | **P value** |
| --- | --- | --- | --- | --- | --- |
| **Primary endpoint** | **Initial Model 3** | **TyG-BMI tertiles** | **5.174** | **2** | **0.0752** |
|  |  | Age | 5.048 | 1 | 0.0247 |
|  |  | Sex | 8.678 | 1 | 0.0032 |
|  |  | LAD | 0.118 | 1 | 0.7307 |
|  |  | Atrial fibrillation | 2.866 | 1 | 0.0905 |
|  |  | **Global test** | **17.137** | **6** | **0.0088** |
|  | **Sex-stratified Model 3** | **TyG-BMI tertiles** | **5.071** | **2** | **0.0790** |
|  |  | Age | 2.405 | 1 | 0.1210 |
|  |  | LAD | 0.020 | 1 | 0.8880 |
|  |  | Atrial fibrillation | 2.987 | 1 | 0.0840 |
|  |  | **Global test** | **9.677** | **5** | **0.0850** |
| **All-cause death** | **Initial Model 3** | **TyG-BMI tertiles** | **5.188** | **2** | **0.0747** |
|  |  | Age | 0.587 | 1 | 0.4435 |
|  |  | Sex | 10.587 | 1 | 0.0011 |
|  |  | LVOTG | 0.594 | 1 | 0.4407 |
|  |  | Atrial fibrillation | 3.010 | 1 | 0.0827 |
|  |  | **Global test** | **17.840** | **6** | **0.0066** |
|  | **Sex-stratified Model 3** | **TyG-BMI tertiles** | **5.069** | **2** | **0.0790** |
|  |  | Age | 0.022 | 1 | 0.8830 |
|  |  | LVOTG | 0.126 | 1 | 0.7230 |
|  |  | Atrial fibrillation | 3.517 | 1 | 0.0610 |
|  |  | **Global test** | **10.138** | **5** | **0.0710** |

LAD, left atrial diameter; LVOTG, left ventricular outflow tract gradient; TyG-BMI, triglyceride-glucose body mass index;
