# Supplementary material for: Association between triglyceride glucose–body mass index and clinical outcomes after septal myectomy
Source: Front Endocrinol (Lausanne). 2026 Jul 30;17:1855510. doi: 10.3389/fendo.2026.1855510 (PMC13467838; doi:10.3389/fendo.2026.1855510)
Supplement: Supplementary file 2 [file Table2.docx]

**Table S2. Univariable Cox regression analysis of primary endpoint and all-cause death**

| Variables | Primary Endpoint | | All-cause death | |
| --- | --- | --- | --- | --- |
|  | HR (95%CI) | P-value | HR (95%CI) | P-value |
| Age (years) | 1.035(1.016-1.054) | <0.001 | 1.037 (1.008-1.067) | 0.013 |
| Sex | 0.826(0.551-1.239) | 0.356 | 1.066(0.573-1.986) | 0.839 |
| NYHA III/IV | 1.050(0.670-1.645) | 0.830 | 0.845(0.439-1.628) | 0.615 |
| CABG | 1.018(0.602-1.721) | 0.948 | 1.130(0.521-2.450) | 0.758 |
| Mitral valve procedure | 1.426(0.833-2.441) | 0.196 | 0.748(0.266-2.098) | 0.581 |
| Diabetes mellitus | 0.875(0.356-2.154) | 0.772 | 1.238(0.382-4.013) | 0.721 |
| Hypertension | 1.152(0.753-1.763) | 0.515 | 1.040(0.538-2.008) | 0.908 |
| Atrial fibrillation | 2.091(1.316-3.321) | 0.002 | 2.513(1.282-4.926) | 0.007 |
| LAD | 1.030(1.003-1.058) | 0.028 | 1.036(0.995-1.078) | 0.084 |
| LVEDD | 1.011(0.974-1.048) | 0.576 | 1.012(0.957-1.070) | 0.670 |
| PWT | 1.065(0.995-1.140) | 0.070 | 1.091(0.991-1.201) | 0.075 |
| LVEF | 0.969(0.939-1.000) | 0.050 | 0.956(0.911-1.002) | 0.062 |
| MWT | 1.023(0.985-1.061) | 0.240 | 1.030(0.974-1.089) | 0.301 |
| Moderate to severe MR | 1.164(0.775-1.748) | 0.464 | 1.235(0.664-2.297) | 0.505 |
| Maximum LVOTG | 0.999(0.992-1.006) | 0.858 | 1.010(1.002-1.019) | 0.022 |
| TyG-BMI | 1.007(1.002-1.013) | 0.004 | 1.012(1.005-1.019) | <0.001 |
| TyG-BMI tertiles |  |  |  |  |
| T1 | Reference | - | Reference | - |
| T2 | 1.70(0.98-2.97) | 0.060 | 2.59(1.01-6.68) | 0.049 |
| T3 | 2.12(1.24-3.61) | 0.006 | 3.37(1.35-8.38) | 0.009 |

BMI = Body Mass Index; LAD = Left Atrial Dimension; LVEDD = Left Ventricular End-Diastolic Dimension; LVEF = Left Ventricular Ejection Fraction; LVOTG = Left Ventricular Outflow Tract Gradient; MR = Mitral Regurgitation; NYHA = New York Heart Association; PWT=Posterior Wall Thickness; RVD= Right Ventricular Dimension;
